# Association between antioxidant vitamins and oxidative stress among patients with a complete hydatidiform mole

**DOI:** 10.6061/clinics/2020/e1724

**Published:** 2020-07-02

**Authors:** Leda Ferraz, Catharina Albiero Bueno Ramos, Antônio Braga, Luis Guillermo Coca Velarde, Kevin M. Elias, Neil S. Horowitz, Patrícia Fátima Lopes, Ross S. Berkowitz

**Affiliations:** I Programa de Pos-Graduacao em Ciencias Medicas, Universidade Federal Fluminense, Niteroi, RJ, BR; II Programa de Pos-Graduacao em Saude Perinatal, Centro de Doencas Trofoblasticas, Maternidade-Escola, Universidade Federal do Rio de Janeiro, Rio de Janeiro, RJ, BR; III Division of Gynecologic Oncology, Department of Obstetrics and Gynecology and Reproductive Biology, New England Trophoblastic Disease Center, Brigham and Women’s Hospital, Dana-Farber Cancer Institute, Boston, MA, USA; IV Departamento de Patologia, Faculdade de Medicina, Universidade Federal Fluminense, Niteroi, RJ, BR

**Keywords:** Complete hydatidiform mole, Gestational trophoblastic disease, Oxidative stress, Antioxidants, Vitamins

## Abstract

**OBJECTIVES:**

This study aimed to evaluate the potential relationship between oxidative stress, dietary intake, and serum levels of antioxidants in patients with a complete hydatidiform mole (CHM) compared with controls.

**METHODS:**

This was an observational cross-sectional study conducted in Rio de Janeiro, Brazil. A total of 140 women were enrolled in this study and divided into four groups: 43 patients with CHM, 33 women who had had an abortion, 32 healthy pregnant women, and 32 healthy non-pregnant women. All participants underwent blood sampling, assessment using a semiquantitative food frequency questionnaire, and anthropometric measurement. Blood samples were collected after overnight fasting (10-12 h). Vitamin levels (A, C, and E) were determined by ultra-performance liquid chromatography, and gamma-glutamyl transferase levels were assessed using an automated quantitative analysis system (Dimension^®^, Siemens).

**RESULTS:**

Although all groups showed sufficient serum vitamin A and E levels, the participants had inadequate dietary intake of these vitamins. Conversely, all groups had an insufficient serum level of vitamin C, despite adequate intake. The gamma-glutamyl transferase values did not differ significantly among the groups. However, elevated serum levels of this enzyme were observed in several patients.

**CONCLUSIONS:**

All groups exhibited high levels of oxidative stress, as evaluated by gamma-glutamyl transferase levels, and had inadequate intake of antioxidant vitamins. Therefore, the high exposure to oxidative stress found in our study, even in healthy pregnant and non-pregnant women, may increase the incidence of CHM in this region.

## INTRODUCTION

Hydatidiform mole (HM) is a benign disorder resulting from an abnormal fertilization. It is characterized by atypical swelling of the placental villi and intense trophoblastic hyperplasia (1). It is the most common form of gestational trophoblastic disease (GTD) and can progress to a malignant condition known as gestational trophoblastic neoplasia (GTN) (2). HM comprises two distinct entities, partial HM and complete HM (CHM), based on clinical, morphological, and genetic characteristics (1,2).

In Brazil, the estimated incidence of HM is 1 per 200-400 pregnancies, which is 5-10 times higher than that in Europe and the United States (0.6-1.1 per 1,000). This discrepancy in the incidence of HM worldwide could be attributed to methodological differences or the lack of centralized databases, or it could be linked to genetic, socioeconomic, and nutritional factors (3). Although higher incidences of HM in developing and underdeveloped countries are often attributed to nutritional deficiencies, no study has thoroughly tested this hypothesis.

The etiology of HM is not yet completely understood (4). However, oxidative stress (OS) may be intimately involved in its pathogenesis, as it leads to deoxyribonucleic acid damage and defective placentation (5,6). High levels of OS markers and decreased levels of antioxidant defense components have been found in CHM patients compared with the levels in healthy pregnant and non-pregnant women (7,8).

The main components of human non-enzymatic antioxidant defense mechanism encompass dietary vitamins A, C, and E and glutathione (GSH). Nutritional deficiencies in these elements are potential risk factors for CHM (3,4). Decreased consumption of carotenoids as well as lower serum levels of vitamins A, D, and E and folate have been reported in HM (8-10). To properly assess nutritional status and antioxidant levels, food intake assessment should be followed by biochemical evaluation of blood samples (11). However, previous studies have not evaluated either the dietary intake or the serum level of antioxidant vitamins A, C, and E as well as GSH in women with CHM and the control groups.

Vitamins A and E are considered important dietary antioxidants because of their ability to reduce oxidative activity in lipophilic environments. Vitamin A is well established as an efficient scavenger of free radicals (12), while vitamin E can prevent lipid peroxidation (13). Vitamin C, on the contrary, has potent antioxidant activity in hydrophilic environments, acting as a scavenger of reactive oxygen and nitrogen species. In addition, vitamin C plays a significant role in the regeneration of oxidized vitamin E (14).

GSH plays a central role in the intracellular non-enzymatic antioxidant system. Besides neutralizing free radicals, GSH is essential in the regeneration of vitamin C (15). The metabolism and homeostasis of GSH are regulated by gamma-glutamyl transferase (GGT). Although GGT has been largely used as a marker of alcohol consumption and hepatobiliary status, some studies have suggested the use of circulating GGT within the reference interval as an early systemic OS marker. Accordingly, levels of GGT increase in response to OS because of the increased need of intracellular GSH for maintenance of adequate redox balance (16). Moreover, increased levels of GGT are strongly correlated with low serum levels of antioxidant vitamins (A, C, and E) (17).

Supplementation with antioxidant vitamins, like A, C, and E, has been suggested as a strategy for the prevention of CHM and its progression to GTN (8,18). However, studies examining the link between OS, dietary intake, and serum levels of antioxidants among patients with CHM are limited. Hence, this study aimed to analyze the serum levels and dietary intake of antioxidant vitamins as well as to evaluate systemic OS by measuring serum GGT levels in patients with CHM to better understand the potential role of nutrition in the pathogenesis of CHM in the Brazilian population.

## MATERIAL AND METHODS

This was an observational cross-sectional study conducted in two Brazilian GTD reference centers (RCs) located in the state of Rio de Janeiro at the Antonio Pedro University Hospital (Fluminense Federal University [UFF]) and the Maternity School (UFRJ). Both RCs are located in public hospitals that are part of the Brazilian Unified Health System.

The study was approved by the Human Research Ethics Committees of the Medical School, UFF (CAAE: 37660714.7.0000.5243) and the Maternity School, UFRJ (CAAE: 38329214.0.0000.5275). All procedures performed were in accordance with the ethical standards of the institutional and/or national research committee as well as the 1964 Helsinki Declaration and its later amendments or comparable ethical standards. Informed consent was obtained from all individual participants included in the study.

### Design and study population

We enrolled 140 women in this study, of whom 43 women had a CHM diagnosis confirmed by histopathological examination (CHM group), 33 women had histopathological examination findings indicative of spontaneous non-molar abortion (abortion, Ab group), 32 were healthy women at 20 weeks of gestation with a single viable fetus (healthy pregnancy, HP group), and 32 were healthy non-pregnant women (non-pregnant, NP group).

Every pregnant woman visiting the RCs who was suspected of having a molar pregnancy and had undergone molar evacuation was invited to participate in the study when they arrived for their first medical appointment after uterine evacuation. After histopathological diagnosis, these patients were allocated to two goups according to their diagnosis: complete hydatidiform mole (CHM) group and non-molar abortion (Ab) group. The patients in the other two groups (HP and NP groups) were recruited in the non-GTD clinics and paired by age with those in the CHM group. For the healthy pregnant group, only participants with a gestational age of up to 20 weeks were recruited.

### Sample collection for biochemical parameters and nutritional assessment

As most cases of uterine evacuation are not elective, the team decided to conduct the recruitment during the first visit to the RC after uterine evacuation. This ensured an overnight fasting period (10 to 12 hours) before venous blood collection, as well as guaranteed a regular dietary intake the day before blood collection. Vitamin levels were determined by ultra-performance liquid chromatography. GGT, aspartate aminotransferase, alanine aminotransferase, and bilirubin were assessed using an automated quantitative analysis system (Dimension^^®^^, Siemens).

The GGT cut-off value for OS has not yet been established (19). In this study, to evaluate GGT as an OS marker, we applied both Yamada et al.’s (19) parameters, which considered GGT elevation as values within the last quartile, and those used in Cho et al. study, which defined GGT elevation as values above the median of the population (15).

All participants underwent nutritional intake assessment and anthropometric measurements, [weight (kg), height (m), and body mass index (BMI, kg/m^2^)], according to Lohman et al. (20). The blood pressure was measured according to the American Heart Association guidelines (21).

For the evaluation of vitamin intake, a semiquantitative food frequency questionnaire (SFFQ) was used. The food list in the SFFQ was determined based on the foods mostly consumed by the Brazilian population (22). The frequency of food intake was recorded in units of time, *i.e.*, “daily,” “weekly,” “monthly,” and “not ingested.” The quantities of food intake estimated using in-home measures were transformed into grams using the 4^th^ edition of the Table for Evaluation of Food Consumption in Household Measures (23). The resulting equivalent intake values of each vitamin were obtained using the nutritional assessment program Avanutri PC^^®^^ (Avanutri, Brazil).

Since the recommended dietary allowance (RDA) for vitamins varies according to age and is increased during gestation, the estimated intake (mg/day) has been converted to intake adequacy (%). The RDAs used to calculate the intake adequacy were as follows: vitamin A=700 µg/day for non-pregnant women, 750 and 770 µg/day of vitamin A for pregnant women (<19 or ≥19 years, respectively), vitamin C=65 mg/day and 75 mg/day for non-pregnant women (<19 or ≥19 years, respectively); 80 mg/day and 85 for pregnant women (<19 or ≥19 years, respectively), and vitamin E=15 mg/day for all participants.

### Statistical analysis

Given that the previous literature has mostly focused on molar pregnancy and low vitamin A intake, the study was powered to see the difference of at least 0.1 mg/L below the lower limit of the vitamin A reference range of 0.3 mg/L. At least 24 women were needed in each group to detect a difference between 0.3±0.1 mg/L and 0.2±0.1 mg/L at 80% power at an alpha level of 0.01. Data were expressed as medians, first and third quartile, or frequencies and percentages, as appropriate. Continuous variables were evaluated for normality using the Shapiro-Wilk test. For variables with normal distribution, the groups were compared using analysis of variance; for variables with non-normal distribution, Kruskal-Wallis test was used followed by a non-parametric Student’s *t*-test or a Mann-Whitney test with Bonferroni correction. The frequencies were compared using the Fisher's exact test and Pearson’s chi-square test as appropriate. All statistical analyses were performed using R version 3.2.2, and *p-*values of <0.05 were considered significant.

## RESULTS

The demographic and laboratory characteristics of the studied population are shown in Table 1. Considering age, race, BMI, systolic and diastolic blood pressure, and vitamin supplementation (A, C, and E) parameters, no statistically significant differences were observed among the groups. Similarly, none of the groups had cases of hepatobiliary dysfunction. Therefore, it can be inferred that, in all groups in this study, GGT reflected systemic OS levels independent of hepatic function.

The median serum levels and intake adequacy of antioxidant vitamins are described in Table 2. The CHM group had significantly higher serum vitamin A levels than the HP group (*p*=0.004). On the contrary, the vitamin A serum levels in the Ab and NP groups were higher than those in the CHM group (*p*=0.018 and *p*=0.046, respectively). Although significant differences were observed among the groups, all groups showed normal median vitamin A serum levels, with serum concentrations within the reference interval (0.3-0.7 mg/dL). Similarly, with regard to intake adequacy, all groups had a median adequate intake of this vitamin, with no significant difference between the groups (*p*=0.215). However, the prevalence rates of vitamin A intake inadequacy were 48.8% in the CHM group, 37.5% in the Ab group, 40.6% in the HP group, and 46.8% in the NP group.

As regards vitamin C, the serum levels were compatible with the classification of deficiency, which means none of the four studied groups achieved the lower limit of the normal range (0.5 mg/dL). Circulating vitamin C deficiency was observed in 71% of the studied population and did not differ significantly among the groups (*p*=0.624). Conversely, all groups showed a median vitamin C intake over 100% with no significant difference between groups (*p*=0.958). However, the prevalence rates of inadequate vitamin C intake were 39.5%, 42.4%, 28.1%, and 34.3%, respectively, in the CHM, Ab, HP, and NP groups.

With regard to vitamin E, all participants showed normal serum levels, with serum concentrations above the reference value for this vitamin (12 μmol/L). No statistically significant difference was found among the four groups (*p*=0.675). However, in terms of the daily vitamin E intake, all groups had a median vitamin E intake adequacy lower than 100%, with no significant difference among the groups (*p*=0.052). Moreover, most patients in all groups did not take enough vitamin E, demonstrating prevalence rates of inadequate dietary intake of 55.8% in the CHM group, 72.7% in the Ab group, 53.1% in the HP group, and 75% in the NP group.

**Figure 1:**
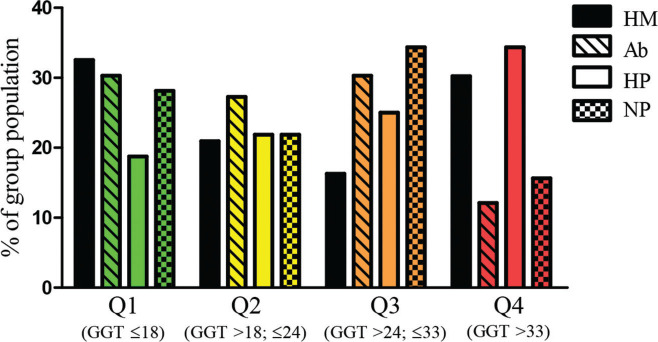
Distribution of each group’s population by percentage according to the gamma-glutamyl transferase (GGT) quartiles (Q1, Q2, Q3, and Q4) (n=140). CHM - Complete hydatidiform mole; Ab - Abortion; HP - Healthy pregnant; NP - Non-pregnant.

When we analyzed the serum GGT levels as an OS marker, the quartiles and median of the studied population were as follows: median: 24 U/L, first quartile (Q1): ≤18 U/L, second quartile (Q2): >18 and ≤24 U/L, third quartile (Q3): >24 and ≤33 U/L, and fourth quartile (Q4): >33 U/L). The population distribution, in percentage, of each studied group distributed into GGT quartiles, is shown in Figure 1. All groups had a similar percentage of participants above the GGT median (*p*=0.559) and Q4 (*p*=0.084), presenting similar exposure to OS.

## DISCUSSION

Pregnancy is characterized by a physiological increase in free radical production and, consequently, increased recruitment of antioxidant defenses. Thus, an adequate supply of antioxidant vitamins in this period is essential to ensure the maintenance of oxidative balance. Moreover, OS plays an important role in the pathogenesis of CHM (5,7,8), and GGT has been increasingly used as an oxidative imbalance marker (15).

Our results on serum vitamin A disagree with those reported by Andrijono et al. (24) and Kolusari et al. (2). They observed a reduction in the circulating levels of vitamin A in women with molar pregnancy compared with those with a healthy pregnance. Meanwhile, we observed an increase in the vitamin A serum levels among healthy pregnant women. When the groups were compared, all participants presented with vitamin A serum levels within the reference range (2). Berkowitz et al. reported a decrease in carotene (pre-vitamin A) intake in women with CHM compared with matched controls (4). However, while that study was primarily conducted in patients and matched controls with private health insurance, the current investigation was performed in free-care public hospitals in the Brazilian Unified Health System. It is important to emphasize the high occurrence of vitamin A intake inadequacy in our studied population. This finding may be explained by the fact that vitamin A is fat soluble. Thus, deficient intake with normal serum levels may indicate that body stores are being mobilized; however, despite the deficient intake, serum vitamin A deficiency will not occur.

Vitamin C, along with vitamin E, comprises the first line of defense and is the most important of the non-enzymatic diet antioxidants (15,17,19). This is the first study to evaluate vitamin C serum levels and intake in patients with CHM. Preeclampsia is a gestational disease that has OS-associated pathogenesis, similar to that suggested for HM. Several studies have observed a reduction in serum vitamin C levels in preeclamptic women compared with healthy pregnant women (25). In our study, however, we found no statistically significant difference in serum vitamin C levels between the groups.

We found a high prevalence of serum vitamin C deficiency in more than 70% of the participants. Oliveira et al. reported 30.8% C-hypovitaminosis in pregnant women from southeastern Brazil, the same region where this study was conducted (27). The high prevalence of serum vitamin C deficiency found is alarming because low serum vitamin C levels are associated with an increased risk of premature rupture of membranes, low birth weight, and preeclampsia, among other adverse outcomes of gestation (25,26). Because vitamin C is a water-soluble vitamin that is not stored in the body, reaching adequate blood vitamin C levels depends on the daily ingestion of at least the RDA. Given that 40% of our population had inadequate daily vitamin C intake, it is not surprising that serum levels of this vitamin were low, thus placing these women at risk of negative effects of vitamin C deficiency. The findings of other studies carried out in Brazil are in agreement with our results since they described a high prevalence of inadequate consumption of vitamin C (28).

With regard to the serum levels of vitamin E, our results disagree with those of Kolusari et al., who reported a decrease in serum levels of vitamin E among patients with CHM (8). We did not find significant differences among the studied groups in terms of vitamin E serum levels. Péter et al. assessed the serum vitamin E levels in adolescents and adults and reported a serum sufficiency of vitamin E worldwide, which is similar to the findings in our study (13).

Vitamin E sufficiency is described as serum concentrations higher than 12 μmol/L; however, many studies have already indicated that optimal levels, greater than 30 μmol/L, have benefits in the prevention of several types of cancers (29,30). In our study, although all participants showed serum vitamin E adequacy, 80.7% (113 participants) did not have the optimal serum levels for this vitamin. Our finding agrees with the literature demonstrating that only 21% of the global population had optimal vitamin E serum levels (13).

However, despite the serum sufficiency and similar to what was described by Péter et al. regarding vitamin ingestion, (13) we observed an inadequacy in vitamin E intake in most participants from all groups. One explanation for this finding is that vitamin E (similar to vitamin A) is fat soluble and can therefore be stored. Nevertheless, the high prevalence of inadequate consumption of vitamin E predisposes these women to future deficiency once their reserves are exhausted (30).

OS has already been associated with the pathogenesis of CHM since higher concentrations of its markers and lower levels of antioxidants have previously been observed in women with this disease (5-8). In addition, antioxidant vitamin supplementation has already been proposed for HM prevention and as a secondary prevention strategy for postmolar GTN (18). However, in contrast to the other reports in the literature, no significant differences in serum concentrations or food intake of antioxidant vitamins A, C, and E were found after comparing the groups. A reduced intake of antioxidant nutrients can contribute to the occurrence of a population profile of high OS, as found in this sample. Because of the high OS pattern found in all groups analyzed in this study, under certain conditions, this microenvironment may favor the occurrence of reproductive anomalies, such as HM.

A novel aspect of the present study is the finding that GGT is a systemic and early OS marker among patients with CHM. Our findings corroborate with those of other studies reporting that elevated levels of GGT are associated with inflammation and subclinical OS in a healthy population (17,19). Thus, the high exposure to OS found in our study, even in healthy non-pregnant women, may contribute to an increase in the incidence of CHM in this region.

This study has some limitations. During the gestational period, the woman undergoes several physiological changes to ensure the development of the fetus, and these metabolic changes are reflected in laboratory tests at the time of blood collection. In contrast, CHM and AB groups blood samples collection were performed after uterine evacuation. This condition may affect the comparison of samples between the groups. Although the samples included in this study were statistically adequate for analyzing vitamin A, it would be appropriate to carry out a more extensive case series evaluation not only to study the parameters analyzed with a higher power but also to evaluate the relationship between nutrition, other vitamins (such as C and E), and the progression of molar pregnancy to GTN. Moreover, patients in all four groups in our study were managed in two public, free-care hospitals, and their nutritional status may differ from those in studies conducted in populations with greater resources (4,10). The strengths of this study include the evaluation of both serum levels and assessment of the intake of antioxidant vitamins among patients with complete molar pregnancy as well as the comparison between healthy pregnant and non-pregnant patients. By broadening the serum evaluation based on the usual dietary intake, we were able to assess not only the condition of the vitamin levels and OS profile at the time of diagnosis of the CHM but also gain insight into the probable vitamin levels at conception, which may be important when considering the role of nutrition in the occurrence of CHM. In addition, this study has provided new information about GGT as a marker of OS in patients with CHM.

## CONCLUSION

While it is believed that OS and nutrition are closely related to the pathogenesis of CHM, other studies are necessary to elucidate this relationship. In our study, nutritional deficiency has been observed equally in women with CHM, as among those with aAb, HP women, and NP healthy women. Therefore, until more extensive results are published, it may not be appropriate to advocate nutritional supplementation strategy specifically as a way to prevent CHM. Given the frequent nutritional deficiencies in the Brazilian population, a healthy diet, especially in those that intend to become pregnant, should be recommended and supported for all women.

## AUTHOR CONTRIBUTIONS

Ramos CAB, Ferraz L, Braga A and Lopes PF conceived and designed the study and were responsible for fulfilling the ethical requirements during the design and execution of the study. Ferraz L was responsible for all nutritional assessments. Ramos CAB and Ferraz L were responsible for conducting the biochemical evaluation, audited by Lopes PF. Velarde LGC was responsible for the sample size calculation and statistical analysis. All authors contributed to the data analysis, interpretation, and manuscript writing and final approval.

## Figures and Tables

**Table 1:** Demographic and laboratory characteristics of the study population (n=140).

Parameters	CHM (n=43)	Ab (n=33)	HP (n=32)	NP (n=32)	*p*-value
Age (years)	25 (19-27.2)	29 (23-37)	25 (19.7-30.5)	30 (23-38.2)	0.068
<20	12 (27.9%)	6 (18.2%)	8 (25%)	2 (6.2%)	0.142
20-40	28 (65.1%)	23 (69.7%)	23 (71.9%)	24 (75%)	
>40	3 (7%)	4 (12.1%)	1 (3.1%)	6 (18.7%)	
Educational level					
Elementary	13 (31.2%)	9 (27.3%)	8 (25%)	1 (3.1%)	0.001**
High school	23 (53.5%)	17 (51.5%)	15 (46.9%)	6 (18.7%)	
College	7 (16.3%)	6 (18.1%)	9 (28.1%)	25 (78.1%)	
Race***					
White	18 (41.9%)	18 (54.5%)	13 (40.6%)	20 (62.5%)	0.566
Brown	16 (37.2%)	10 (30.3%)	13 (40.6%)	7 (21.9%)	
Black	9 (20.9%)	5 (15.2%)	6 (18.8%)	5 (15.6%)	
BMI (kg/m^2^)	24.3 (21.7-27)	25.6 (22.9-28.1)	24.8 (22-27.2)	23 (21.4-27.7)	0.625
SBP (mmHg)	106.7 (100.8-113.8)	105.6 (100-113.3)	107 (96-109.6)	105.5 (101.3-113.2)	0.805
DBP (mmHg)	67.3 (63.8-71.8)	68 (64.3-74)	68.75 (61.2-74.1)	69.8 (64.5-75.3)	0.553
Vitamin A supplementation	2 (4.6%)	3 (9%)	3 (9.3%)	3 (9.3%)	0.819
Vitamin C supplementation	4 (9.3%)	4 (12.1%)	3 (9.3%)	3 (9.3%)	0.980
Vitamin E supplementation	2 (4.6%)	4 (12.1%)	4 (12.5%)	4 (12.5%)	0.541
AST (RI: 15-37 U/L)	22 (19-27.5)a	23 (19-28)a	19 (16.7-22)b	19 (16-2)b	0.004
ALT (RI: 14-59 U/L)	21 (15.5-29)	19 (1-24)	21 (17-31.5)	23 (18.7-31.2)	0.284
TB (RV <1.1 mg/dL)	0.7 (0.4-0.7)a	0.7 (0.5-0.8)a	0.3 (0.2-0.4)b	0.5 (0.3-0.6)c	<0.001
DB (RV <0.3 mg/dL)	0.3 (0.2-0.4)a	0.3 (0.3-0.5)b	0.08 (0.06-0.11)c	0.1 (0.09-0.14)d	<0.001
IB (RV <0.8 mg/dL)	0.3 (0.2-0.4)a	0.3 (0.2-0.4)*	0.2 (0.2-0.3)b	0.3 (0.2-0.5)c*	0.014

Notes: Data are expressed as median (first and third quartiles) and frequencies (%).

CHM - Complete hydatidiform mole. Ab - Abortion. HP - Healthy pregnant. NP - Non-pregnant. BMI - Body mass index. SBP - Systolic blood pressure. DPB - Diastolic blood pressure. AST - Aspartate aminotransferase. ALT - Alanine aminotransferase. TB - Total bilirubin. IB - Indirect bilirubin. DB - Direct bilirubin. RV - Reference value. RI - Reference interval. Continuous variables were compared using Kruskal-Wallis test, while categorical variables were compared using Fisher’s exact test.

* Different letters and indicate significant differences (*p*<0.05).

** CHM *vs* Ab *p*=0.159; CHM *vs* HP *p*=0.078; Ab *vs* HP *p*=0.113.

*** Race was classified as self-reported skin color.

**Table 2:** Median values of serum levels and estimated intake of vitamins A, C, and E in the studied population (n=140).

Vitamins		CHM (n=43)	Ab (n=33)	HP (n=32)	NP (n=32)	*p*-value
Vitamin A	Serum level (RI 0.3-0.7 mg/L)	0.4 (0.4-0.5)a	0.5 (0.4-0.6)b	0.4 (0.3-0.5)c	0.4 (0.4-0.6)b	0.004
	Intake adequacy, %	115.4 (44.6- 203.9)	211.5 (55.3-280.8)	141 (72.8-312.5)	110.1 (67.3-217.6)	0.215
Vitamin C	Serum level (RI 0.5-1.5 mg/dL)	0.2 (0.1-0.4)	0.3 (0.1-0.5)	0.3 (0.2-0.5)	0.3 (0.1-0.5)	0.624
	Intake adequacy, %	124.9 (78.2-280.3)	142.3 (77.1-255.5)	153.2 (90.7-246.2)	128 (82.1-254.8)	0.958
Vitamin E	Serum level, (RV >12 µmol/L)	23 (18.4-27.3)	24.1 (21.7-28.2)	23.9 (20.5-29.7)	23.1 (20.4-28.2)	0.675
	Intake adequacy, %	90.6 (65.6-139.3)	79.3 (66.6-101.3)	98.3 (67.8-116)	63 (29.5-95.6)	0.052

Notes: Data are expressed as median (first and third quartiles).

CHM - Complete hydatidiform mole. Ab - Abortion. HP - Healthy pregnant. NP - Non-pregnant. RV - Reference value. RI - Reference interval. Variables were compared using Kruskal-Wallis test. Different letters represent significate statistical differences (*p*<0.05). Vitamin A serum levels *p*-values: CHM *vs* Ab=0.018; CHM *vs* HP=0.004; CHM *vs* NP=0.046.
